# Association between tomato consumption and prehypertension among Korean adults: finding from the Korean Genome and Epidemiology Study

**DOI:** 10.1017/S0007114525105710

**Published:** 2026-01-28

**Authors:** Wuttyi Khaing, Dogyeong Kim, Hyojeong Kim, Eunjae Cho, Kyungjoon Lim, Sangah Shin

**Affiliations:** 1 Department of Food and Nutrition, Chung-Ang University, Gyeonggi-do, South Korea; 2 Faculty of Medicine and Health, School of Medical Sciences, University of Sydney, Sydney, Australia

**Keywords:** Diet, Tomato consumption, Hypertension, Prehypertension

## Abstract

A significant association between tomato consumption and a lower risk of developing hypertension has been reported. In this study, we aimed to investigate the relationship between tomato intake and prehypertension risk among Korean adults. Hypertension was defined according to the criteria established by the Korean Society of Hypertension. The study participants were selected from the Health Examinees cohort study. Tomato consumption was measured using an FFQ and categorised into quintiles based on the amount consumed. Higher tomato consumption was associated with a lower risk of prehypertension in men (hazard ratio (HR) 0·86, 95 % CI 0·80, 0·92, *P*_for trend_ 0·0005). Women in the highest quintile also showed a similar trend (HR 0·94, 95 % CI 0·90, 0·99, *P*_for trend_ 0·0091). Stratified analyses revealed a reduced risk of prehypertension across all subgroups, except underweight individuals and those with a history of alcohol consumption (all *P*_for interaction_ < 0·05). These findings indicate that higher tomato intake may offer potential advantages for managing blood pressure levels.

Hypertension has become a major and increasingly prevalent health concern worldwide. Data from 2023 indicated that approximately 29·4 % of Korean adults aged ≥ 20 years, or approximately 12·6 million individuals, had hypertension in 2020. The prevalence of diagnosed cases more than tripled between 2002 and 2020. Correspondingly, the number of individuals using antihypertensive medication increased nearly 4-fold, while adherence increased more than 12-fold^(1)^. Hypertension, defined as a systolic blood pressure (SBP) of 140 mmHg or higher, or a diastolic blood pressure (DBP) of 90 mmHg or higher^(2)^, is affected by various lifestyle and dietary factors. Notably, high Na consumption significantly impacts hypertension^(3)^. The WHO recommends a daily Na intake of < 2000 mg, but the average Na consumption among Koreans is 3477·2–3889·6 mg per d. Choosing high-Na foods, such as kimchi and instant noodles, contributes to increased Na levels^(4)^. Hence, managing risk factors for developing hypertension is essential among Korean adults.

Tomatoes contain several vitamins, minerals, fibre and carbohydrates and are associated with various positive health effects. Their antioxidant potential is mainly owing to lycopene compounds, which possess beneficial effects at different stages of atherosclerosis. Lycopene influences serum lipid levels, endothelial dysfunction, inflammation, blood pressure and antioxidant potential^(5)^. Additionally, the potassium in tomatoes improves endothelial and smooth muscle function^(6)^. Research has consistently demonstrated that adequate intake of potassium helps regulate blood pressure to more desirable levels. The blood pressure-lowering effects of potassium have been observed in various intervention trials and reported in meta-analyses^(7,8)^.

In 2022, a study found an inverse relationship between tomato intake and the risk of developing hypertension^(9)^. Another study demonstrated that supplementation with 250 mg of lycopene-rich tomato extract significantly reduces SBP and DBP among patients with grade 1 hypertension, indicating that the antioxidant and vasodilating properties of lycopene may contribute to improved blood pressure regulation^(10)^. However, limited research has investigated the relationship between tomato consumption and hypertension risk, particularly among Korean adults. Given the evidence suggesting that lycopene and potassium, both abundant in tomatoes, may help lower blood pressure^(5,6)^, analysing the relationship between tomato consumption and hypertension is essential in this population.

As the prevalence of hypertension among Koreans continues to increase, its treatment and management have received considerable attention. Prehypertension has recently been identified as a potential predictor of clinical hypertension and, by extension, an indicator of increased cardiovascular risk^(11)^. Therefore, in this prospective cohort study, we aimed to investigate the relationship between tomato consumption and the prevalence of prehypertension in Korean adults.

## Methods

### Study population

**Figure 1.**
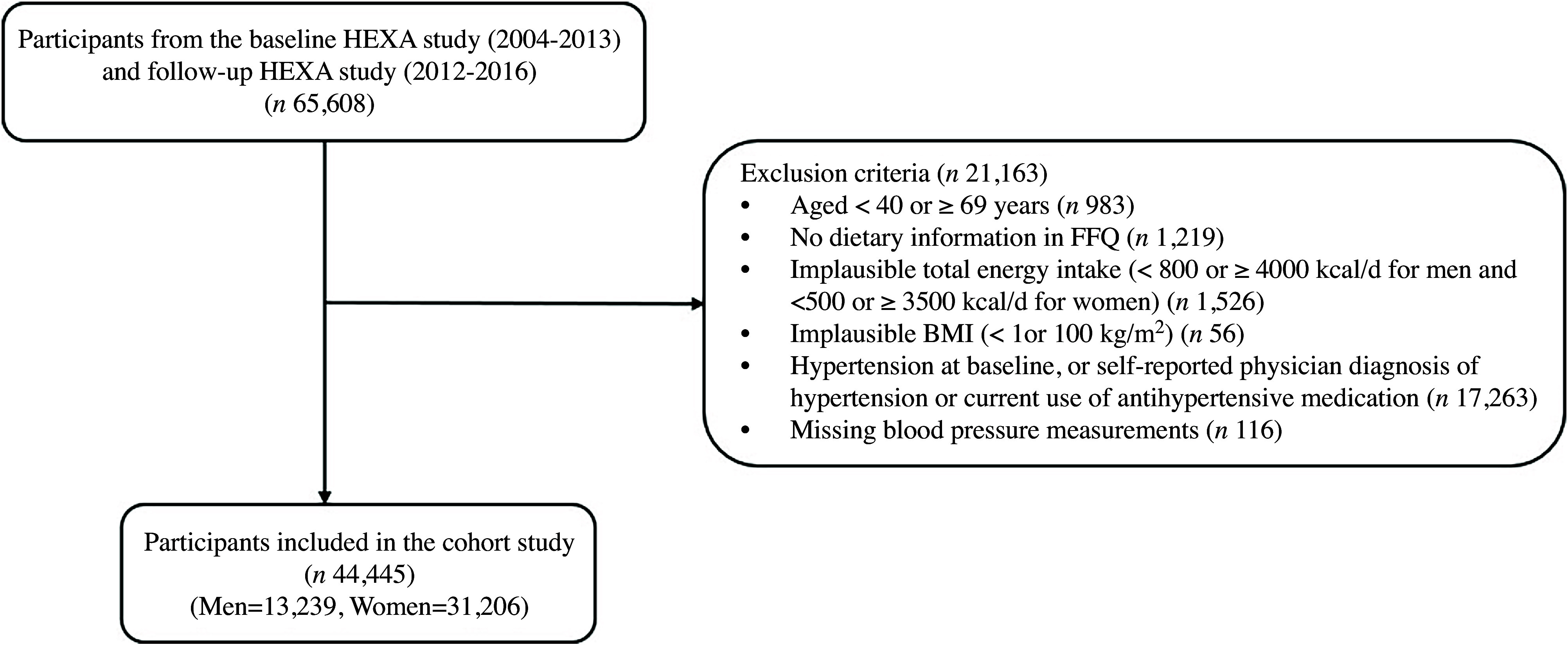
Flow chart of study participants from the Health Examinees (HEXA) study in Korea.

Participants were selected from the Health Examinees study (HEXA), part of the Korean Genome and Epidemiology Study (KoGES), which is a large-scale genomic cohort study designed to investigate the epidemiological and genetic characteristics of chronic diseases in Korea^(12)^. From 2004 to 2013, 173 195 participants aged 40–79 years attended thirty-eight general hospitals and health screening centres across eight regions in Korea for initial interviews and measurements. Of these, 65 608 participants completed the follow-up survey between 2012 and 2016. For this study, 21 163 participants were excluded under the following criteria: those younger than 40 years or older than 69 years (*n* 983); those without dietary information from the FFQ (*n* 1219); those with implausible energy intake (< 800 or ≥ 4000 kcal/d for men and < 500 or ≥ 3500 kcal/d for women) (*n* 1526); those with implausible BMI (< 1 or > 100 kg/m^2^) (*n* 56); those with hypertension at baseline, or self-reported physician diagnosis of hypertension or current use of antihypertensive medication (*n* 17 263); and those with missing blood pressure measurements (*n* 116). This yielded a final sample of 44 445 participants (13 239 men and 31 206 women) (Figure 1).

### Dietary data assessment

Tomato intake was assessed using a validated semi-quantitative FFQ comprising 106 food items. Participants reported how often and how much of each food they had consumed during the previous year. Consumption frequency was categorised into nine levels ranging from ‘never or seldom’ to ‘three times per day’. The average daily intake of raw tomatoes, cherry tomatoes and tomato juice was estimated for each participant. To reduce random within-person variation, this average daily intake was calculated across the two surveys. The KoGES FFQ has been validated against twelve 3-d diet records collected over four seasons, with energy-adjusted correlation coefficients ranging from 0·23 (vitamin A) to 0·64 (carbohydrate) and a median of 0·39 across nutrients, and reproducibility assessed 1 year apart showing a median correlation of 0·45 for nutrient intakes^(13)^. Participants were then categorised into sex-specific quintiles (Q1–Q5), with Q1 representing the lowest and Q5 the highest. Median values were calculated for each quintile and assigned to participants to allow tests of linear trend across categories in regression analyses. Tomato intake was analysed as unadjusted (absolute) values, with total energy intake included as a covariate in multivariable models. In addition, sensitivity analyses were performed using the residual method (online Supplementary Table 1).

### Definition of hypertension and prehypertension

Blood pressure (SBP and DBP) was measured twice using standardised mercury sphygmomanometers, and the average of the two readings was used in the analysis. According to the Korean Society of Hypertension, hypertension is defined as SBP of 140 mmHg or higher or DBP of 90 mmHg or higher. Prehypertension was defined as SBP ranging from 130 to 139 mmHg or DBP from 80 to 89 mmHg, in accordance with the same guidelines. In addition, participants who reported a physician diagnosis of hypertension or current use of antihypertensive medication were also classified as hypertensive^(14)^.

### Covariate variables

Sociodemographic and lifestyle factors that are known to influence hypertension were included as covariates: age, sex, household income, education level, alcohol consumption, smoking status, BMI, energy intake and physical activity. Information on these variables was collected at baseline through self-reported questionnaires, apart from BMI, which was calculated from the measured height and weight. Age was categorised into three groups: 40–49 years, 50–59 years and 60–69 years. Household income was categorised as < 3 million won and ≥ 3 million won. Education level was divided into three groups: below middle school, high school and over college. Alcohol consumption was categorised as non-drinker, past drinker and current drinker. Smoking status was categorised as never smoker, past smoker and current smoker. BMI categories were underweight (< 18·5 kg/m^2^), normal (18·5–22·9 kg/m^2^), overweight (23·0–24·9 kg/m^2^) and obese (≥ 25·0 kg/m^2^)^(15)^. Energy intake classifications were > 800 kcal/d to < 4000 kcal/d for men and > 500 kcal/d to < 3500 kcal/d for women. Physical activity was categorised as active or inactive.

### Statistical analysis

All analyses were conducted using SAS version 9.4 (SAS Institute). Differences in general participant characteristics according to tomato consumption levels were assessed using χ^2^ tests for categorical variables and general linear regression for continuous variables. Cox proportional hazards regression models were used to estimate hazard ratios (HR) and 95 % CI for the association between tomato consumption and the risk of prehypertension. Tomato intake was analysed both as categorical variables (quintiles of daily intake, with the lowest quintile as reference) and as a continuous variable (per 1 sd increase in intake).

Multivariable models were adjusted sequentially: model 1 was unadjusted; model 2 was adjusted for age and BMI; and model 3 was further adjusted for income, education, alcohol consumption, smoking status, physical activity and total energy intake. Tests for linear trend (P_for trend_) were conducted by modelling the median value of each consumption category as a continuous variable in the Cox regression. To assess potential effect modification, stratified analyses were conducted according to age (< 53 years and ≥ 53 years), BMI (< 23·0 kg/m^2^ and ≥ 23·0 kg/m^2^), alcohol consumption status (non-drinker, past drinker and current drinker) and smoking status (never smoker, past smoker and current smoker). The statistical significance level was set at *P* < 0·05. Interaction terms between tomato intake and each stratifying variable were added to the multivariable Cox models, and the statistical significance of interaction was evaluated using the likelihood ratio test (*P*_for interaction_). The proportional hazards assumption was assessed by examining log(−log) survival curves across exposure categories and by including time-dependent covariates, with no meaningful violations observed.

## Results

A total of 44 445 participants (13 239 men and 31 206 women) were included in the analysis. Table 1 presents the general characteristics of participants based on tomato consumption levels. Increased tomato consumption was observed among women, as well as among those with higher household income and higher educational levels. Physical inactivity was prevalent among those with higher tomato consumption in both men and women (*P* < 0·0001). Among men, higher tomato consumption correlated with lower alcohol consumption and a higher likelihood of being a non-smoker (*P* < 0·0001).

**Table 1.** Baseline characteristics of participants according to the quartiles of tomato consumption

	Tomato consumption										
	Q1		Q2		Q3		Q4		Q5		*P*
Men (*n* 13 239)	3302		2984		2643		2284		2026		
	Mean	sd	Mean	sd	Mean	sd	Mean	sd	Mean	sd	
Age (years)	52·33	8·22	53·24	8·26	53·9	7·96	54·06	7·86	54·67	7·89	< 0·0001
	*n*	%	*n*	%	*n*	%	*n*	%	*n*	%	
Age, *n* (%)											< 0·0001
40s	1296	39·25	1051	35·22	821	31·06	679	29·73	558	27·54	
50s	1233	37·34	1144	38·34	1098	41·54	968	42·38	831	41·02	
60s	773	23·41	789	26·44	724	27·39	637	27·89	637	31·44	
	Mean	sd	Mean	sd	Mean	sd	Mean	sd	Mean	sd	
BMI (kg/m^2^)	24·05	2·69	23·97	2·64	23·97	2·49	23·98	2·56	24·01	2·43	0·7245
	*n*	%	*n*	%	*n*	%	*n*	%	*n*	%	
Obesity, *n* (%)											0·4042
Underweight	52	1·57	55	1·84	33	1·25	32	1·4	19	0·94	
Normal	1079	32·68	1002	33·58	905	34·24	753	32·97	676	33·37	
Overweight	1037	31·41	924	30·97	836	31·63	745	32·62	664	32·77	
Obese	1134	34·34	1003	33·61	869	32·88	754	33·01	667	32·92	
Income level, *n* (%)											< 0·0001
< 3 million won	1502	45·49	1371	45·95	1171	44·31	1052	46·06	812	40·08	
≥ 3 million won	1507	45·64	1368	45·84	1275	48·24	1091	47·77	1097	54·15	
Education level, *n* (%)											< 0·0001
Below middle school	745	22·56	650	21·78	508	19·22	400	17·51	293	14·46	
High school	1395	42·25	1174	39·34	1071	40·52	877	38·4	770	38·01	
Over college	1118	33·86	1128	37·8	1038	39·27	989	43·3	948	46·79	
Alcohol consumption, *n* (%)											< 0·0001
Non-drinker	624	18·9	626	20·98	635	24·03	535	23·42	471	23·25	
Past drinker	214	6·48	205	6·87	174	6·58	158	6·92	159	7·85	
Current drinker	2453	74·29	2141	71·75	1830	69·24	1584	69·35	1389	68·56	
Smoking status, *n* (%)											< 0·0001
Never smoker	772	23·38	826	27·68	802	30·34	704	30·82	688	33·96	
Past smoker	1207	36·55	1220	40·88	1139	43·09	1008	44·13	882	43·53	
Current smoker	1316	39·85	926	31·03	696	26·33	569	24·91	452	22·31	
Physical activity, *n* (%)											< 0·0001
Active	2291	69·93	1939	65·37	1629	61·85	1312	57·85	1066	52·96	
Inactive	985	30·07	1027	34·63	1005	38·15	956	42·15	947	47·04	
Women (*n* 31 206)	5608		5891		6220		6693		6794		
	Mean	sd	Mean	sd	Mean	sd	Mean	sd	Mean	sd	
Age (years)	51·15	7·44	51·32	7·40	51·57	7·27	51·63	7·21	51·62	6·97	0·0003
	*n*	%	*n*	%	*n*	%	*n*	%	*n*	%	
Age, *n* (%)											< 0·0001
40s	2454	43·76	2515	42·69	2542	40·87	2723	40·68	2659	39·14	
50s	2290	40·83	2433	41·3	2700	43·41	2895	43·25	3114	45·83	
60s	864	15·41	943	16·01	978	15·72	1075	16·06	1021	15·03	
	Mean	sd	Mean	sd	Mean	sd	Mean	sd	Mean	sd	
BMI (kg/m^2^)	23·27	2·85	23·31	2·78	23·22	2·7	23·17	2·69	23·04	2·58	< 0·0001
	*n*	%	*n*	%	*n*	%	*n*	%	*n*	%	
Obesity, *n* (%)											< 0·0001
Underweight	162	2·89	122	2·07	136	2·19	159	2·38	149	2·19	
Normal	2633	46·95	2763	46·9	2933	47·15	3265	48·78	3459	50·91	
Overweight	1466	26·14	1581	26·84	1723	27·7	1773	26·49	1828	26·91	
Obese	1347	24·02	1425	24·19	1428	22·96	1496	22·35	1358	19·99	
Income level, *n* (%)											< 0·0001
< 3 million won	2725	48·59	2848	48·34	2868	46·11	3085	46·09	2860	42·1	
≥ 3 million won	2165	38·61	2320	39·38	2681	43·1	2998	44·79	3315	48·79	
Education level, *n* (%)											< 0·0001
Below middle school	2064	36·8	2149	36·48	2024	32·54	1937	28·94	1645	24·21	
High school	2492	44·44	2569	43·61	2824	45·4	3170	47·36	3224	47·45	
Over college	980	17·48	1107	18·79	1295	20·82	1532	22·89	1871	27·54	
Alcohol consumption, *n* (%)											< 0·0001
Non-drinker	3636	64·84	3883	65·91	4307	69·24	4491	67·1	4584	67·47	
Past drinker	101	1·8	103	1·75	94	1·51	98	1·46	122	1·8	
Current drinker	1845	32·9	1879	31·9	1799	28·92	2082	31·11	2053	30·22	
Smoking status, *n* (%)											< 0·0001
Never smoker	5301	94·53	5669	96·23	6049	97·25	6518	97·39	6575	96·78	
Past smoker	84	1·5	69	1·17	55	0·88	80	1·2	83	1·22	
Current smoker	184	3·28	123	2·09	93	1·5	74	1·11	100	1·47	
Physical activity, *n* (%)											< 0·0001
Active	3796	68·36	3846	65·92	3885	63·04	3947	59·46	3757	55·59	
Inactive	1757	31·64	1988	34·08	2278	36·96	2691	40·54	3002	44·41	

Table 2 shows the HR and 95 % CI for prehypertension according to quintiles of tomato consumption. Participants with higher tomato consumption exhibited a trend towards better blood pressure levels than those with lower intake. After adjusting for all covariates, the highest quintile of tomato consumption was associated with a lower risk of prehypertension in men (HR: 0·86, 95 % CI 0·80, 0·92, *P*_for trend_: 0·0005) and women (HR: 0·94, 95 % CI 0·90, 0·99, *P*_for trend_: 0·0091). When we analysed as a continuous variable, each 1-sd increase in tomato intake was associated with a lower risk of prehypertension in both men (HR 0·96, 95 % CI 0·93, 0·98) and women (HR 0·97, 95 % CI 0·96, 0·99) in the fully adjusted model. These findings were consistent with results from the quintile-based analyses. Notably, in women, the association was not evident in the crude model but became significant after multivariable adjustment, suggesting potential confounding by lifestyle factors.

**Table 2.** HR and 95 % CI for prehypertension according to tomato consumption

	Tomato consumption (g/d)											
	Q1	Q2		Q3		Q4		Q5		*P*_for trend_	Per sd increment	
		HR	95 % CI	HR	95 % CI	HR	95 % CI	HR	95 % CI		HR	95 % CI
Men (*n* 13 239)	3302	2984		2643		2284		2026				
Median (g/d)	4·17	10·71		19·64		32·14		66·07				
Range (g/d)	0·00–7·08	7·29–14·88		15·0–25·6		25·83–44·64		44·94–750·0				
Person-year												
Mean	4·9	5·0		4·9		4·8		4·9				
Sum	16173·9	14911·6		13025·8		11041·1		9954·6				
Prehypertension												
Case, *n*	2216	1966		1718		1473		1259				
Model 1	1·00 (Ref.)	0·92	0·86, 0·97	0·94	0·88, 0·99	0·97	0·91, 1·04	0·90	0·84, 0·96	0·0486	0·97	0·94, 0·99
Model 2	1·00 (Ref.)	0·91	0·86, 0·97	0·93	0·88, 0·99	0·96	0·90, 1·03	0·89	0·83, 0·96	0·0236	0·96	0·94, 0·99
Model 3	1·00 (Ref.)	0·91	0·85, 0·97	0·92	0·86, 0·98	0·93	0·87, 0·99	0·86	0·80, 0·92	0·0005	0·96	0·93, 0·98
Women (*n* 31 206)	5608	5891		6220		6693		6794				
Median (g/d)	4·17	10·71		19·64		32·14		69·64				
Range (g/d)	0·00–7·08	7·29–14·88		15–25·60		25·83–44·64		44·94–800·0				
Person-year												
Mean	5·0	5·1		5·0		5·0		5·0				
Sum	28175·5	29757·7		31061·5		33180·0		34133·0				
Prehypertension												
Case, *n*	2781	2933		3062		3409		3271				
Model 1	1·00 (Ref.)	1·00	0·95, 1·06	1·03	0·98, 0·88	1·08	1·03, 1·13	1·00	0·95, 1·05	0·7771	0·98	0·96, 0·99
Model 2	1·00 (Ref.)	1·00	0·95, 1·06	1·03	0·98, 1·09	1·07	1·02, 1·13	1·00	0·95, 1·06	0·8847	0·98	0·97, 0·99
Model 3	1·00 (Ref.)	1·00	0·95, 1·05	1·00	0·95, 1·06	1·02	0·97, 1·07	0·94	0·90, 0·99	0·0091	0·97	0·96, 0·99

HR, hazard ratios.

*n* 44 445; values are presented as *n*, median (min-max) and HR (95 % CI).

Model 1: Crude model.

Model 2: Adjusted for age and BMI.

Model 3: Additionally adjusted for education level, income level, smoking status, alcohol consumption and total energy intake; *P* values for trends were calculated using multivariable Cox proportional hazards regression.

**Figure 2.**
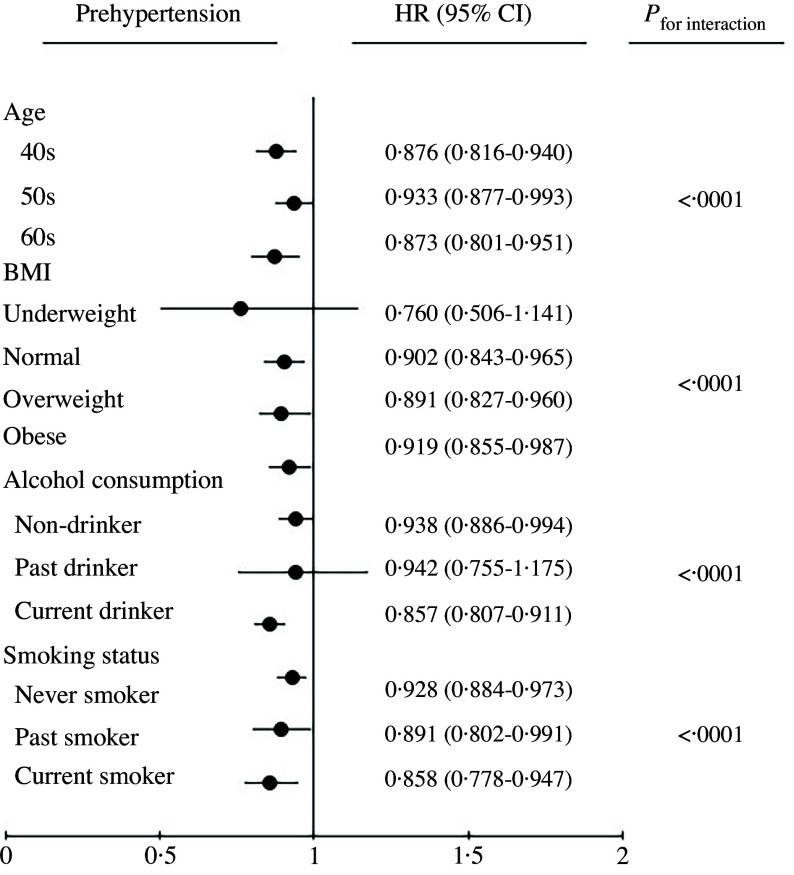
HR and 95 % CI for prehypertension in the comparison of the highest quintile of tomato consumption as stratified covariates. Values are presented as HR (95 % CI). Non-drinker: never consumed alcohol; past drinker: previously consumed alcohol but no longer does; current drinker: currently consumes alcohol. Never smoker: never smoked cigarettes; past smoker: previously smoked > 400 cigarettes but no longer does; current smoker: currently smokes and has smoked > 400 cigarettes in their lifetime. HR, hazard ratio.

Stratified analyses were also conducted to assess the association between tomato consumption and prehypertension across subgroups based on age, BMI, alcohol consumption status and smoking status as shown in Figure 2. The protective effect of higher tomato consumption was significant in most subgroups, except for the underweight group and past drinkers (all *P*_for interaction_ < 0·05).

## Discussion

This comprehensive cohort study analysed the relationship between tomato consumption and prehypertension risk in a large sample of Korean adults. Findings revealed that higher tomato intake was associated with a reduced risk of prehypertension in both men and women, even after adjusting for potential confounders. These findings are consistent with those reported by previous studies highlighting the cardiovascular benefits of tomato consumption and its bioactive components. For instance, a cohort study of 11 460 Chinese adults aged > 18 years reported a 49–58 % lower risk of new-onset hypertension in those who were in the tomato-fed group than those in the non-fed groups^(9)^. In addition, recent studies have shown that higher consumption of tomatoes and lycopene is significantly associated with reduced risks of total mortality, CHD and cerebrovascular mortality, suggesting the potential health benefits of incorporating these foods into a balanced diet^(16)^. In animal studies, mice supplemented with tomato products and lycopene exhibited reduced blood sugar levels, favourable inflammatory responses and enhanced metabolite profiles linked to antioxidant activity^(17,18)^. These beneficial effects may be contributed to the presence of beneficial constituents, such as lycopene, flavonoids and ascorbic acid. Lycopene, one of the most potent antioxidants and the most commonly found carotenoid in human plasma^(19)^, is presumed to be an active compound responsible for the advantage of tomato consumption^(20)^. However, excessive tomato consumption may weaken their protective effects, which are associated with excessive solanine consumption^(9)^.

The blood pressure-lowering properties of tomatoes and lycopene are known to be due to stimulating nitric oxide production in endothelial cells^(21)^. Daily consumption of tomato extract or juice for at least 4 weeks significantly reduces blood pressure^(22,23)^. Oxidative stress plays a key role in hypertension by generating reactive oxygen species that impair vascular function, leading to reduced nitric oxide synthesis and diminished antioxidant bioavailability^(24)^. Animal studies have shown that long-term lycopene consumption effectively protects against oxidative stress-induced DNA damage in the liver^(25)^. In addition, data from the National Health and Nutrition Examination Survey indicated an inverse relationship between plasma lycopene levels and hypertension prevalence in overweight and obese individuals^(26)^. In addition to lycopene, tomatoes are rich in hydrophilic compounds particularly polyphenols, such as flavonoids and phenolic acids, which modulate hypertension risk through various mechanisms^(27,28)^. A randomised controlled trial found that these hydrophilic compounds beneficially lower blood pressure in men with prehypertension by inhibiting angiotensin-converting enzyme activity^(22)^. Potassium also contributes to blood pressure reduction, and 100 g of tomatoes contains 212 mg of potassium, helping to prevent the harmful effects of Na^(29)^.

A key strength of this study is its use of large cohort data and extended follow-up periods among Korean adults to examine the association between tomato consumption and hypertension. The use of a validated FFQ, standardised blood pressure measurements and extensive covariate adjustment further strengthened the reliability of our findings. In addition, the large and geographically diverse sample enhances the generalisability of the results to middle-aged and older Korean adults. However, the limitations should be considered. First, the study did not distinguish between raw and processed tomato products, which may have differing health effects owing to added Na or other ingredients. Second, nutrient-level variables such as lycopene and potassium were not included in our analysis, which limited direct evaluation of their specific contributions. Further studies are needed to disentangle these effects by incorporating nutrient-level covariates or biomarker data, such as plasma lycopene and serum potassium levels. Although such analyses were beyond the scope of the present dataset, further research with detailed nutrient composition or biomarker information will help clarify whether the observed protective association is attributable to specific components or to the overall food matrix of tomato consumption. Finally, a considerable number of participants were excluded because of missing or implausible dietary data and pre-existing hypertension; however, these exclusions were determined by predefined and objective criteria to ensure validity of the measurements. Such exclusion approaches are widely applied in large-scale cohort studies^(30)^. While some degree of selection bias cannot be completely ruled out, the final analytic sample was sufficiently large and broadly representative of middle-aged and older Korean adults.

The study’s findings emphasise the potential of dietary interventions, particularly increased tomato consumption, as a strategy to prevent prehypertension and hypertension. This observation is pertinent in countries like Korea, where high Na intake poses significant public health challenges. Future research should include longitudinal dietary assessments and examine the distinct effects of raw *v*. processed tomato products to provide more precise dietary recommendations.

In conclusion, this study contributes to the increasing evidence that higher tomato consumption is associated with a reduced risk of prehypertension. Incorporating tomatoes into a balanced diet may provide significant benefits for blood pressure management and could be promoted as a preventive measure to mitigate the progression to clinical hypertension.

## Supporting information

Khaing et al. supplementary material Khaing et al. supplementary material

## Supplementary material

For supplementary material/s referred to in this article, please visit https://doi.org/10.1017/S0007114525105710
