# Supplementary material for: Association between tomato consumption and prehypertension among Korean adults: finding from the Korean Genome and Epidemiology Study
Source: Br J Nutr. 2025 Nov 19;135(2):194–200. doi: 10.1017/S0007114525105710 (PMC12885870; doi:10.1017/S0007114525105710)
Supplement: Khaing et al. supplementary material [file S0007114525105710sup001.docx]

Supplementary Table 1. Association between tomato intake (residual energy-adjusted) and risk of prehypertension.

|  | **Residual adjusted tomato intake** | | | | |  |
| --- | --- | --- | --- | --- | --- | --- |
|  | Q1 | Q2 | Q3 | Q4 | Q5 | p for trend |
| **Men (n = 13,239)** | 2,647 | 2,648 | 2,648 | 2,648 | 2,648 |  |
| Median (range), g/day | 1.43 (-21.79-5.53) | 8.71 (5.53-11.97) | 15.78 (11.97-20.50) | 26.71 (20.50-35.63) | 54.92 (35.63-729.20) |  |
| Person year, mean (sum) | 4.9 (12970.3) | 5.0 (13222.0) | 5.0 (13115.2) | 4.9 (12889.9) | 4.9 (12909.6) |  |
| **Prehypertension** | |  |  |  |  |  |
| Case, n | 1,805 | 1,770 | 1,709 | 1,690 | 1,658 |  |
| Model 1 | 1.00 (Ref.) | 0.92 (0.87-0.99) | 0.91 (0.85-0.97) | 0.94 (0.88-1.00) | 0.92 (0.86-0.98) | 0.0886 |
| Model 2 | 1.00 (Ref.) | 0.93 (0.87-0.99) | 0.91 (0.85-0.97) | 0.93 (0.87-1.00) | 0.91 (0.85-0.98) | 0.0644 |
| Model 3 | 1.00 (Ref.) | 0.94 (0.88-1.00) | 0.91 (0.85-0.97) | 0.92 (0.86-0.99) | 0.89 (0.83-0.95) | 0.0034 |
|  |  |  |  |  |  |  |
| **Women (n = 31,206)** | 6,241 | 6,241 | 6,242 | 6,241 | 6,241 |  |
| Median (range), g/day | 5.44 (-23.85-9.99) | 14.12 (10.00-18.38) | 28.39 (18.39-29.10) | 36.24 (29.10-47.96) | 71.82 (47.96-806.34) |  |
| Person year, mean (sum) | 5.1 (31579.5) | 5.0 (31342.4) | 5.0 (31196.8) | 4.9 (30668.0) | 5.1 (31521.0) |  |
| **Prehypertension** | |  |  |  |  |  |
| Case, n | 3,078 | 3,091 | 3,155 | 3,153 | 2,979 |  |
| Model 1 | 1.00 (Ref.) | 1.04 (0.99-1.09) | 1.08 (1.03-1.14) | 1.13 (1.07-1.18) | 1.00 (0.95-1.05) | 0.6767 |
| Model 2 | 1.00 (Ref.) | 1.02 (0.97-1.07) | 1.05 (1.00-1.11) | 1.09 (1.04-1.15) | 0.98 (0.94-1.03) | 0.4257 |
| Model 3 | 1.00 (Ref.) | 1.01 (0.96-1.06) | 1.03 (0.98-1.08) | 1.02 (0.97-1.08) | 0.92 (0.88-0.97) | 0.0002 |

Note: N = 44445; Values are presented as n, median (min-max), and hazard ratios (95% confidence intervals).

Model 1: Crude model.

Model 2: Adjusted for age and body mass index.

Model 3: Additionally adjusted for education level, income level, smoking status, and alcohol consumption; p values for trends were calculated using multivariable Cox proportional hazards regression.
